# Gut Microbiota Patterns in Patients with Non-Alcoholic Fatty Liver Disease: A Comprehensive Assessment Using Three Analysis Methods

**DOI:** 10.3390/ijms242015272

**Published:** 2023-10-17

**Authors:** Anna V. Korobeinikova, Olga A. Zlobovskaya, Anna F. Sheptulina, German A. Ashniev, Maria M. Bobrova, Adel A. Yafarova, Dariga U. Akasheva, Shuanat Sh. Kabieva, Siroj Yu. Bakoev, Anjelica V. Zagaynova, Maria V. Lukashina, Ivan A. Abramov, Mariya S. Pokrovskaya, Yurii V. Doludin, Larisa R. Tolkacheva, Alexander S. Kurnosov, Elena V. Zyatenkova, Evgeniya A. Lavrenova, Irina A. Efimova, Evgeniya V. Glazunova, Anton R. Kiselev, German A. Shipulin, Anna V. Kontsevaya, Anton A. Keskinov, Vladimir S. Yudin, Valentin V. Makarov, Oxana M. Drapkina, Sergey M. Yudin

**Affiliations:** 1 Centre for Strategic Planning and Management of Biomedical Health Risks of Federal Medical Biological Agency, Pogodinskaya Str., 10/1, 119121 Moscow, Russia; akorobeinikova@cspfmba.ru (A.V.K.); shkabieva@cspfmba.ru (S.S.K.); sbakoev@cspfmba.ru (S.Y.B.); mlukashina@cspmz.ru (M.V.L.); akurnosov@cspfmba.ru (A.S.K.);; 2 National Medical Research Center for Therapy and Preventive Medicine, Petroverigskyj Lane 10, bld.3, 101990 Moscow, Russia; asheptulina@gnicpm.ru (A.F.S.); aiafarova@gnicpm.ru (A.A.Y.); dakasheva@gnicpm.ru (D.U.A.);

**Keywords:** non-alcoholic fatty liver disease, bacterial cultures, gut microbiome, qPCR, metagenome, 16S rRNA

## Abstract

Non-alcoholic fatty liver disease (NAFLD) is considered the most common chronic liver disease worldwide, affecting nearly 25% of the global adult population. Increasing evidence suggests that functional and compositional changes in the gut microbiota may contribute to the development and promote the progression of NAFLD. 16S rRNA gene next-generation sequencing is widely used to determine specific features of the NAFLD microbiome, but a complex system such as the gut microbiota requires a comprehensive approach. We used three different approaches: MALDI-TOF-MS of bacterial cultures, qPCR, and 16S NGS sequencing, as well as a wide variety of statistical methods to assess the differences in gut microbiota composition between NAFLD patients without significant fibrosis and the control group. The listed methods showed enrichment in *Collinsella* sp. and *Oscillospiraceae* for the control samples and enrichment in *Lachnospiraceae* (and in particular *Dorea* sp.) and *Veillonellaceae* in NAFLD. The families, *Bifidobacteriaceae*, *Lactobacillaceae*, and *Enterococcaceae* (particularly *Enterococcus faecium* and *Enterococcus faecalis*), were also found to be important taxa for NAFLD microbiome evaluation. Considering individual method observations, an increase in *Candida krusei* and a decrease in *Bacteroides uniformis* for NAFLD patients were detected using MALDI-TOF-MS. An increase in *Gracilibacteraceae*, *Chitinophagaceae*, *Pirellulaceae*, *Erysipelatoclostridiaceae*, *Muribaculaceae*, and *Comamonadaceae*, and a decrease in *Acidaminococcaceae* in NAFLD were observed with 16S NGS, and enrichment in *Fusobacterium nucleatum* was shown using qPCR analysis. These findings confirm that NAFLD is associated with changes in gut microbiota composition. Further investigations are required to determine the cause-and-effect relationships and the impact of microbiota-derived compounds on the development and progression of NAFLD.

## 1. Introduction

Nowadays, non-alcoholic fatty liver disease (NAFLD) is considered the most common chronic liver disease worldwide, affecting nearly 25% of the global adult population [1]. Moreover, the prevalence of NAFLD is expected to increase rapidly in the near future, associated with the rise in obesity and aging of the population. There are several important issues concerning NAFLD that may have a great impact on health and the economic burden of the disease. First, NAFLD includes a spectrum of clinical and histological features, ranging from simple steatosis to non-alcoholic steatohepatitis (NASH), with possible progression to cirrhosis and hepatocellular carcinoma (HCC). Secondly, HCC can develop *de novo* in patients with NASH without the absence of liver cirrhosis. However, the annual cumulative incidence of NASH-related HCC is low (2.6%) compared with viral HCC (nearly 4%) [2]. Thirdly, it is now well documented that NAFLD is linked to other conditions common to insulin resistance, such as abnormal lipid levels, metabolic syndrome [3], type 2 diabetes [4], cardiovascular diseases [5], and central nervous system disorders [6]. It is also associated with an increased risk of developing certain extrahepatic cancers, especially gastrointestinal (GI) cancers, breast cancer, and gynecological cancers [7].

The other major medical and biological problem is the insufficient understanding of the morphological and functional basis of NAFLD pathogenesis to predict its course and outcomes. The pathogenesis of NAFLD is considered complex and includes the interaction between genetic, metabolic, inflammatory, and environmental factors. Additionally, increasing evidence suggests that functional and compositional changes in the gut microbiota may contribute to the development and promote the progression of NAFLD [8,9].

Specific consistent microbiome signatures discriminating healthy individuals from those with NAFLD have been described in human studies. For example, it was demonstrated that Proteobacteria were consistently enriched in steatosis and non-alcoholic steatohepatitis, whereas in cirrhosis, the invasion of oral bacteria (genera *Prevotella* or *Veillonella*) into the distal intestine was observed [10,11]. At the same time, some authors [10] indicate that metabolic confounding factors for dysbiosis (which are not always considered) are often present in NAFLD patients; thus, bacterial signatures (genera *Clostridium* and *Lactobacillus*) may overlap between NAFLD and metabolic diseases (type 2 diabetes mellitus). Other factors influencing gut microbiota composition in patients with NAFLD include, but are not limited to, geographical region, ethnicity, population characteristics, microbiome sequencing tools, NAFLD diagnostic tools, disease spectrum, drug consumption, and circadian rhythm. Consequently, these factors should also be considered when planning the research and adjusted during the data analysis [10].

In our study, we aimed to perform a comprehensive comparative analysis of gut microbial patterns in patients with NAFLD without significant fibrosis and patients from the control group. We performed careful patient selection by excluding those suffering from type 2 diabetes mellitus, impaired glucose tolerance, or arterial hypertension, and those following restrictive diets. To obtain comprehensive data reflecting microbiota changes in NAFLD, we used different evaluation methods: bacterial cultures, quantitative polymerase chain reaction (qPCR), and next-generation sequencing (NGS) of the 16S rRNA gene (V3–V4). Our study included only Moscow residents, thus minimizing the effect of geographic region on gut microbiota composition.

## 2. Results

### 2.1. MALDI-TOF-MS

A pairwise comparison was made using the Mann–Whitney test for the NAFLD/control groups. Four bacteria were found to significantly differ between the groups. The results are presented in Table 1.

**Figure 1:**
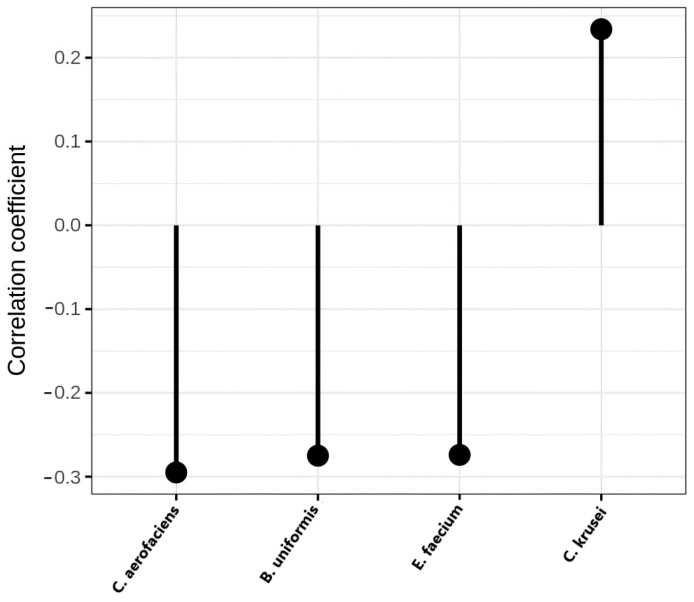
Correlation coefficient for MALDI-TOF-MS data.

The differences in the identified microorganisms were visualized (Figure 1); a negative correlation coefficient indicates enrichment in the control group and a positive correlation coefficient in the NAFLD samples.

**Figure 2:**
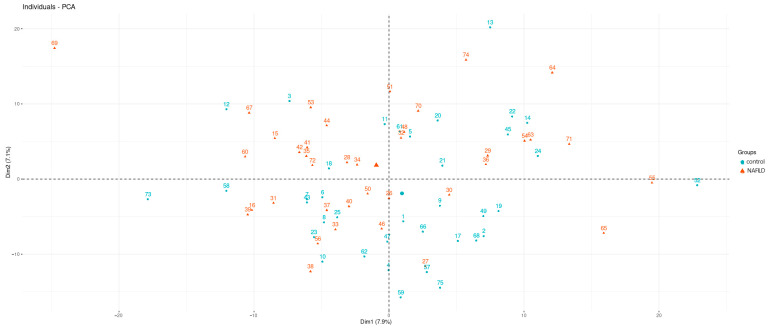
The first two components of the PCA analysis MALDI-TOF-MS.

Principal component analysis was performed to check whether the two groups split into distinct clusters. Based on the visualization of the first two components (Figure 2), we can say that there was no obvious separation of the groups.

The synthesized data were subjected to a quality check, with a final quality score of 99%. The resulting table was used to generate the xgboost model (accuracy = 97%). We selected the 10 most significant predictors, which are presented in Supplementary Figure S2.

### 2.2. Real-Time qPCR Analysis

#### 2.2.1. Data Preprocessing

In order to increase the amount of data for analysis, we tested whether we could combine the PF20, PF21, and FS20 groups (from different collection years and DNA extraction methods, see Section 4). Thus, it was first necessary to ensure that there were no differences between the samples (separately for the control and NAFLD). To solve this, principal component analysis was used. Based on the graphical representation of the data distributions, there were no clear divisions between groups PF20 and PF21. For a more accurate analysis, a pairwise comparison of the two groups’ taxa content was carried out using the Mann–Whitney test. As a result, the content of *Bacteroides* sp. in NAFLD patients and *Clostridium leptum* in the controls differed significantly between the PF20 and PF21 groups. These bacteria were excluded from the subsequent «case-control» analysis.

Linear discriminant analysis (with LOOCV cross-validation) was further performed to verify the results separately for the NAFLD and control samples. As the predictive ability of the model was low, we concluded the absence of significant differences between the compared groups and combined the groups PF20 and PF21 into one for the further analysis (the PF group further on).

Since there were 2–4 significantly different bacteria between samples of the FS20 group (hereafter referred to as the FS group) and the PF group, and the overall estimate of the predictive ability of the LDA model was about 0.7 for both the control and NAFLD, the samples isolated using different methods were further analyzed separately.

#### 2.2.2. Statistical Testing using the Mann–Whitney Test

A pairwise comparison was conducted using the Mann–Whitney test for NAFLD/control samples for groups extracted with two methods. For the PF extraction group, *Collinsella* sp. was significantly more abundant in the control sample (*p* = 0.04 after FDR correction). For the FS extraction group, *Fusobacterium nucleatum* and *Dorea* sp. were significantly more abundant in the NAFLD sample (*p* = 0.021 and *p* = 0.035 after FDR correction, respectively).

#### 2.2.3. Machine Learning Model

The XGBOOST model was built on the synthesized PCR data to detect significant bacteria.

The PF group demonstrated 95% model accuracy. The most important predictors were *Ruminococcus* sp. (enrichment in the control), *Bacteroides* sp., and *Lactobacillaceae* (enrichment in NAFLD) (see Supplementary Figure S3).

The FS group demonstrated 98% model accuracy. The most important predictors were *Bacteroides* sp. (enrichment in the control) and *Fusobacterium nucleatum* (enrichment in NAFLD) (see Supplementary Figure S4).

#### 2.2.4. Correlations within Case/Control Groups

Correlations between bacteria and the parameters of age and BMI were calculated for each sample using Spearman’s method since the data were not normally distributed.

When evaluating the NAFLD group, the PF21+ group containing only NAFLD samples with increased BMI was combined the NAFLD sample from the PF group (group 1 + 3). The FS group was evaluated separately. The results obtained are presented below in the form of correlation matrices (see Supplementary Figures S5–S8).

A number of bacteria showed significant correlations in both samples—both for patients and for controls (see Table 2).

The FS group demonstrated stronger correlations than the PF group (see Supplementary Figures S7 and S8).

Accordingly, more bacteria showed significant correlations in both the NAFLD and control samples when analyzing the FS group, including several correlations with age and BMI (see Table 3).

### 2.3. NGS

**Figure 3:**
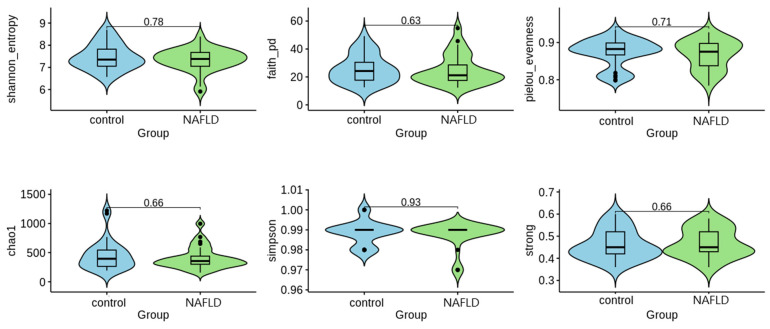
Results of the statistical test for alpha diversity values. The *p*-value scores are presented above each line.

Alpha diversity is a measure of how varied a single sample is, usually taking into account the number of different species observed. In order to access sample richness and/or evenness, six alpha diversity indices were calculated: Shannon, Faith, Pielou, Chao1, Simpson, and Strong (Figure 3). A nonparametric Mann–Whitney statistical test was used to compare the values. Based on the results, we cannot reject the null hypothesis that there are no differences between the groups.

**Figure 4:**
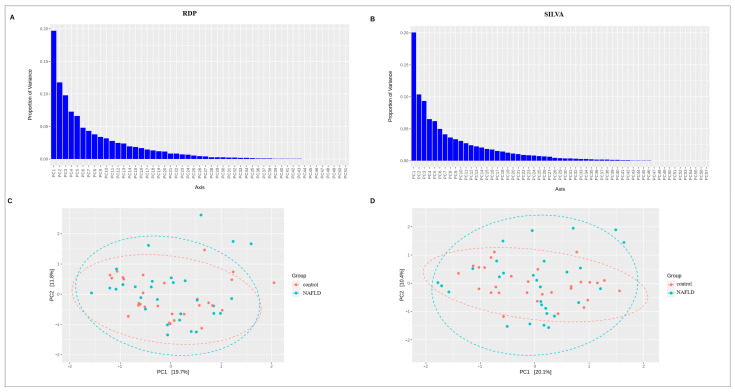
The results of PCA analysis for NGS data. (**A**,**B**) Proportion of variance of RDP and SILVA data, correspondingly; (**C**,**D**) Visualization of the first two components for RDP and SILVA data, correspondingly. The dotted lines indicate the 95% confidence interval.

Beta diversity provides a measure of the similarity or dissimilarity of one microbial composition to another. Eitchison’s distance was used to calculate beta diversity. The graphs below show the main PCA components (Figure 4), followed by a visualization of the first two components. The color marker indicates the location of the groups relative to each other.

**Figure 5:**
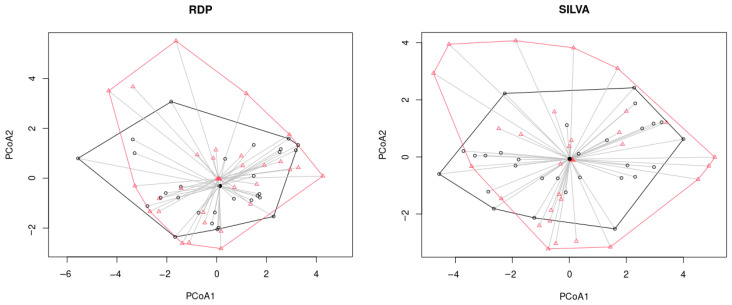
The results of PERMANOVA analysis for NGS data (black line and circles correspond to the control samples, red line and triangles correspond to the NAFLD samples).

We also tested whether the samples cluster using PERMANOVA multivariate analysis of variance. The results for the two methods were similar, with the groups overlapping for the most part (Figure 5).

**Figure 6:**
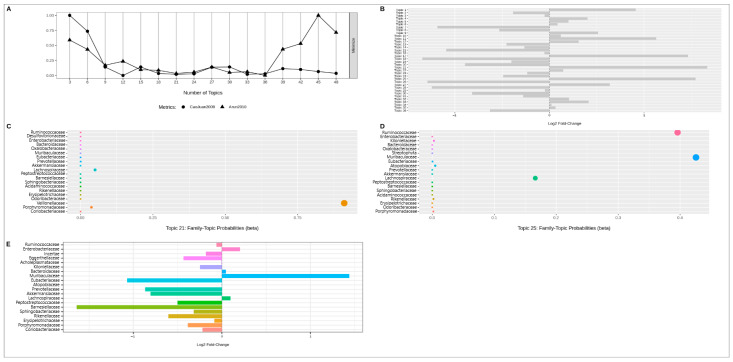
The results of latent Dirichlet allocation analysis for RDP data; (**A**)—Indicator chart for selecting the number of topics; (**B**)—Bacterial topic contribution to sample distinction; (**C**)—Summary of the 21 topics; (**D**)—Summary of the 25 topics; (**E**)—Bacteria contribution to sample distinction. Different colors on the subfigures (**C**–**E**) indicate different bacterial taxa. The dot size on the subfigures (**C**,**D**) reflects the probability value.

The next step was the latent Dirichlet allocation analysis. The number of topics for analysis was determined by the point on the graph where the “CaoJuan2009” and “Arun2010” indicators had the lowest value. For RDP data, the optimal number of topics was 36 (Figure 6A). Of the topics obtained, #21 and #25 showed the greatest contribution to the difference of samples in probability (~1.5 times, Figure 6B). It is worth noting that topics 21 and 25, although showing a numerical difference, did not pass the threshold of significance (using FDR) when refining the p-value (*p*-adj > 0.05). Graphs C and D on Figure 6 show the proportions of probability of bacterial representation in the identified topics, with *Veillonellaceae* (bac69, abundance in NAFLD, topic 21) and *Ruminococcaceae* (bac10, abundance in control), *Muribaculaceae* (bac25, abundance in NAFLD), *Lachnospiraceae* (bac31, abundance in NAFLD) (topic 25) being the most represented. Graph E represents the association of each family individually with NAFLD status. *Muribaculaceae* (bac25, abundance in NAFLD) and *Barnesiellaceae* (bac41, abundance in control) showed up at this stage.

**Figure 7:**
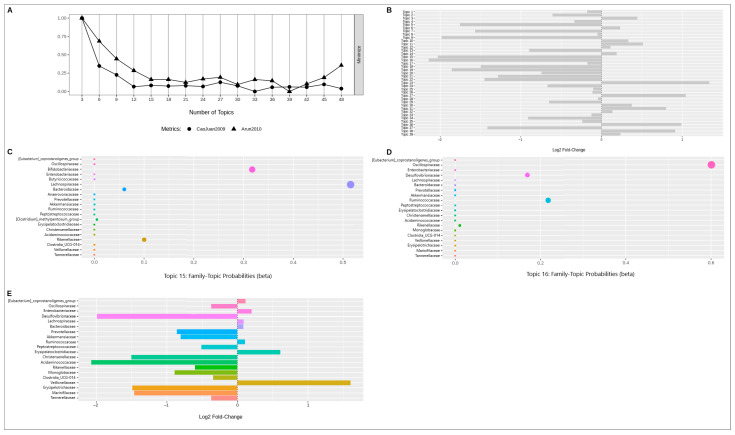
The results of latent Dirichlet allocation analysis for SILVA data; (**A**)—Indicator chart for selecting the number of topics; (**B**)—Bacterial topic contribution to sample distinction; (**C**)—Summary of the 21 topics; (**D**)—Summary of the 25 topics; (**E**)—Bacteria contribution to sample distinction. Different colors on the subfigures (**C**–**E**) indicate different bacterial taxa. The dot size on the subfigures (**C**,**D**) reflects the probability value.

The same procedure was reproduced for the SILVA data (Figure 7).

In this case, the data themes were divided into 39 topics (Figure 7A). The largest differences were shown by topics 15 and 16; however, the topics did not pass the threshold of significance (*p*-adj > 0.05). The following families had greater contributions: *Lachnospiraceae* (bac17, abundance in NAFLD), *Bifidobacteriaceae* (bac133, abundance in control; topic 15) and *Oscillospiraceae* (bac10, abundance in control), *Ruminococcaceae* (bac4, abundance in NAFLD) and *Desulfovibrionaceae* (bac145, abundance in control) (topic 16). When looking at the contribution of each family separately, the difference between the samples was evident for *Desulfovibrionaceae* and *Acidaminococcaceae* (bac 59, abundance in control).

**Figure 8:**
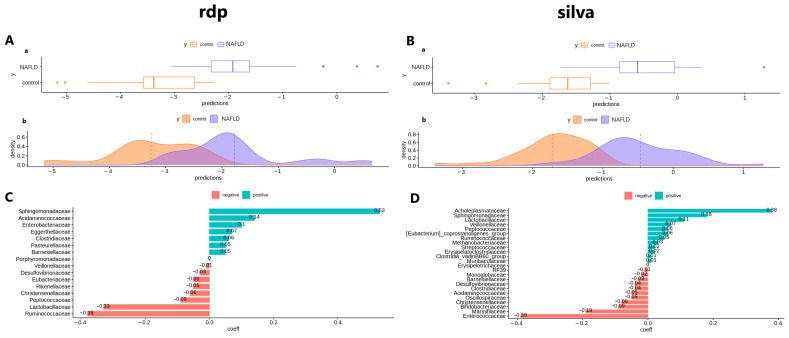
The results of penalty regression for NGS data; (**A**)—Sample distribution of RDP data ((**a**)—box plot and (**b**)—density plot of the data); (**B**)—Sample distribution of SILVA data ((**a**)—box plot and (**b**)—density plot of the data); (**C**)—Contribution coefficients of bacteria to sample difference (RDP data); (**D**)—Contribution coefficients of bacteria to sample difference (SILVA data).

Microbial signatures were further identified using penalty regression. A graphical representation of the taxa composing the signature and their coefficients is presented in Figure 8.

Figure 8A,B graphs confirm the presumed absence of a normal distribution in the data. The families, *Sphingomonadaceae* (abundance in NAFLD), *Ruminococcaceae* (abundance in control), and *Lactobacillaceae* (abundance in control), showed the greatest contribution in the analysis of the RDP results, whereas the families, *Acholeplasmataceae* (abundance in NAFLD) and *Enterococcaceae* (abundance in control) showed the greatest contribution in the analysis of the SILVA results.

Further, the Mann–Whitney test was used to compare the NAFLD and control samples. The results, taxonomy, and abundance of the bacteria are presented in Table 4 and Table 5.

### 2.4. Comparative Analysis of Several Assessment Methods

The combination of several assessment methods (PCR, cultures, NGS) and several statistical tools made it possible to obtain a comprehensive picture and comparison of the NAFLD and control sample microbiota. Table 6 below lists the taxonomic groups that were found to be significant for at least two methods of analysis (for example, PCR analysis and the culture method, or two different statistical analyses within the same methodology).

## 3. Discussion

### 3.1. Single Method Results

According to recent research, the gut–liver axis contributes significantly to the pathogenesis and development of NAFLD. Moreover, there are changes in the composition of the intestinal microbiota of NAFLD patients. Various mechanisms correlating the gut microbiota with NAFLD have been proposed, including dysbiosis-induced dysregulation of endothelial barrier function, which allows the translocation of bacterial metabolites and cell wall components to systemic circulation, leading to inflammation [12].

In this research, we determined the differences in gut microbiota composition between NAFLD patients and a control group using MALDI-TOF-MS analysis of cultivated microorganisms, qPCR of selected taxa, and 16S NGS sequencing.

One feature of NAFLD patients determined through the culture method was increased abundance of *Candida krusei* (Mann–Whitney test, *p* < 0.05). It was demonstrated that abundance of *Candida* yeasts in the gut microbiome may be associated with the pathogenesis of NASH via fructose-dependent endogenous alcohol and triglyceride synthesis [13]. Accordingly, in this study, the serum levels of triglycerides in NAFLD patients were increased compared with controls.

MALDI-TOF-MS analysis also showed the abundance of *Bacteroides uniformis, Enterococcus faecium,* and *Collinsella aerofaciens* in the gut microbiome of the control group. It was shown that the members of *Bacteroides* are the main acetic acid-producing bacteria [14], and increased *Collinsella* sp. abundance may impact human epithelial cell proliferation and improve intestinal barrier integrity via short-chain fatty acid (SCFA) production [15]. Several studies have shown that SCFAs affect the progression of NAFLD [16,17]. Therefore, several *Bacteroides* strains, including *B. uniformis*, have been proposed as novel probiotics (pre-clinical trials) to reduce BMI and triglyceride levels [18]. Likewise, enhanced immune defense mechanisms in macrophages and dendritic cells and reduced gut inflammatory signals were observed after oral consumption of *Bacteroides uniformis* CECT 7771 in high-fat diet treated mice, which showed less hepatic fat deposition compared with the control group [19]. Exposure to probiotics, prebiotics, or synbiotics has been shown to improve the liver histology in murine models of NAFLD. Further knowledge about the interactions between dysbiosis, environmental factors, and diet and their influence on the gut–liver axis is required to improve the treatment of NAFLD and related diseases [20].

For qPCR data, three taxa were significantly different between the NAFLD and control samples. *Collinsella* sp. Was significantly more abundant in the control sample (PF group). *Fusobacterium nucleatum* and *Dorea* sp. were enriched in NAFLD (FS group). *Collinsella* sp. colonizes mucosal surfaces [21] and produces vitamin B6 [22]. It may also produce butyric acid [23], but its role and content in NAFLD is controversial [24,25,26]. Similarly, the data on the content of *Dorea* sp. in the gut microbiota of NAFLD patients are contradictory [27,28,29,30,31], though more evidence suggests its abundance in NAFLD. Less conflicting data can be found concerning the *Fusobacterium nucleatum*, a gut opportunistic pathogen causing chronic inflammation [32]. Its association with NAFLD has also been previously shown [33,34].

The differences in gut microbiota composition between NAFLD patients and the control group were also shown through NGS data analysis. For both databases, a decrease in the levels of *Acidaminococcaceae* members was shown in the NAFLD group. Decreased prevalence of *Acidaminococcaceae* is associated with a decrease in SCFA synthesis (valeric and propionic acids), and as a consequence, with the disturbance in the cholesterol metabolism [35]. The NAFLD group demonstrated increased prevalence of *Gracilibacteraceae* and *Erysipelatoclostridiaceae*, which has been reported in earlier observations [36], as well as an increased prevalence of *Chitinophagaceae* (all observations for the RDP database). The increase in *Gracilibacteraceae* family members is associated with pathological status and systemic inflammation [37]. The *Chitinophagaceae family* has been previously reported to be related to liver diseases and their progression [38].

### 3.2. Cross-Method Comparative Analysis

The combination of several assessment methods (qPCR, cultures, NGS) and several statistical tools made it possible to obtain a comprehensive picture of the gut microbiota in NAFLD and controls and to perform the comparison between samples. In the “Comparative analysis of several assessment methods” section above, Table 6 lists the taxonomic groups that were found to be significant for at least two methods of analysis (for example, PCR analysis and the culture method, or two different statistical analyses within the same methodology).

Thus, when comparing the microbiota of NAFLD and conditionally healthy patients, the most interesting representatives are *Collinsella* sp. (enrichment in controls), *Lachnospiraceae* (and in particular *Dorea* sp.; enrichment in NAFLD), *Oscillospiraceae* (enrichment in controls), and *Veillonellaceae* (enrichment in NAFLD; and in particular *Veillonella* sp.—enrichment in controls). It is worth noting that *Veillonellaceae* is associated with significant fibrosis in non-obese subjects [39].

The families, *Bifidobacteriaceae, Lactobacillaceae*, and *Enterococcaceae* (particularly *Enterococcus faecium* and *Enterococcus faecalis*), were also found to differ significantly between the groups, although their relative abundance either in the NAFLD or control groups was dependent on the method of analysis. In relation to international practice, it is generally accepted that the genus, *Bifidobacterium*, and the family *Bifidobacteriaceae*, are rather associated with NAFLD [40,41,42,43]. The family, *Lactobacillaceae* [28,42] and the genus *Lactobacillus* sp. [30,44], were also more common among NAFLD patients, although contradictory data are also published [40]. Concerning the family, *Enterococcaceae*, there are mainly mentions of an increase in family members and in particular, in the genus, *Enterococcus*, in NAFLD [42,45]. Thus, the above representatives of the microbiota are indeed important in the context of NAFLD, but further studies are needed to establish their use for diagnostic purposes.

### 3.3. Bacterial Correlations

In addition to the analysis of association between the content of certain gut microbiota taxa and NAFLD, we assessed the correlations of various bacterial taxa with each other. In the “qPCR results” section above, Table 2 and Table 3 list all the commonly found associations for both samples within each isolation method. Moreover, we found the general correlations reproducible between isolation methods for one or for both samples (control and/or NAFLD). Table 7 presents these correlations as well as the published data demonstrating the general dynamics for the detected pairs (there were no articles devoted specifically to the analysis of correlations between different bacteria).

For the culture method, there were no significant matching bacteria correlations between the control and NAFLD samples (so that at least one Spearman’s coefficient was more than 0.5). However, when pooling culture data to the same taxonomic levels as those used in qPCR, weak (>0.3) correlations were found between PCR and culture data for two taxa, *Enterobacteriaceae* and *Enterococcus faecalis*, in both samples (control and NAFLD). Besides, for both culture (control and NAFLD) and qPCR (control), a weak positive correlation was observed between these taxa. The increase in some specific groups of conditionally pathogenic and harmful bacteria, including *Enterobacteriaceae* and *Enterococcus faecalis*, has been associated with chronic tissue inflammation [67]. Numerical data are presented in the Table 8.

It should be noted that in contrast to molecular biological methods, the culture method is capable of evaluating only viable organisms, which can lead to a difference in verdicts. This property is both an advantage, since the method provides unique information about a sample, and a disadvantage, since it imposes strict requirements on the conditions for biomaterial collection and storage. Another limitation is that only a small fraction of the gut microbiota can be cultivated, even within the same family. In addition, the culture method can only conditionally be called quantitative, since its accuracy is within the order of magnitude (due to the tenfold dilutions used).

The 16S NGS method is best applied at the family level, with at least 150–200 reads per taxon in the sample to avoid statistical bias for groups with low representation (i.e., about 1% of the total bacterial count in this study, with 19,000 reads per sample). This is a valuable explanatory method; however, due to the complexity of the preparation and analysis stages, this technique has limitations in the accuracy of quantitative assessment.

In contrast, the qPCR method is highly effective for individual quantitative assessment of the microbiota, especially at the level of species and genera. However, it is not an exploratory analysis since it is not able to fully assess the diversity of the community. qPCR is especially recommended in the case of organisms with low representation since it is able to reliably detect and quantify 1–10 genomes of an individual taxon against a total bacterial count of up to 10^8^ in the reaction.

Thus, it is necessary to evaluate the data on the representation of various taxonomic groups by the maximum number of available methods, since each one can explain a separate aspect of the observed clinical picture and partially compensate for the disadvantages of the other methods.

## 4. Materials and Methods

### 4.1. Cohort Description

The study was conducted in accordance with the guidelines of the Declaration of Helsinki and was approved by the Institutional Ethics Committee of the National Medical Research Center for Therapy and Preventive Medicine (protocol No 03-04/20, 28 April 2020; No. 03-07/21, 18 March 2021).

All data were obtained from a prospective cohort of NAFLD patients from the Clinical and Diagnostic Division of the National Medical Research Center for Therapy and Preventive Medicine between March 2020 and December 2021, for whom the inclusion criteria for this study were met. Healthy volunteers comprised the control group.

The NAFLD cohort inclusion criteria were as follows: age from 18 to 75 years, Fatty Liver Index (FLI) of 60 or greater, as well as ultrasound findings consistent with fatty liver (see below), and a signed informed consent form. For the control group, the following inclusion criteria were applied: age from 18 to 75 years, absence of fatty liver disease, sensu FLI values < 60 and ultrasound data, and a signed informed consent form.

Participants with excessive ethanol consumption (>7 and >14 beverages per week for females and males, respectively) were excluded from the study. Other exclusion criteria were as follows: causes of secondary hepatic fat accumulation (medication, Wilson’s disease, viral infections, starvation, or parenteral nutrition); pregnancy and breastfeeding; a history of bariatric surgery, liver cirrhosis and/or hepatocellular carcinoma; extrahepatic malignancy(s) within the last 5 years; morbid obesity; type 1 or 2 diabetes mellitus; chronic obstructive pulmonary disease; bronchial asthma; acute infectious diseases; exacerbation of chronic non-communicable diseases (within four weeks prior to participation in the study); chronic kidney disease stage IIIB or more severe (GFR < 30 mL/min/1.73 m^2^); following restrictive diets; and individuals about to undergo or recovering from a surgical or otherwise medical procedure. Similar exclusion criteria were applied to the control group.

All participants underwent interviewing, physical examination, collection of blood and stool samples, and ultrasound examination of the abdominal cavity. All the above-mentioned procedures were carried out on the same visit.

The initial sample consisted of 199 subjects. Of these, 155 had NAFLD and 44 belonged to the control group. The main characteristics of the participants are presented in Table 9.

Human stool samples were donated by the patients and healthy volunteers who signed informed consent, in accordance with the local ethics committee. Immediately after delivery, the stool samples were frozen at −80 °C until use.

### 4.2. Microbial Identification using MALDI-TOF-MS

For relevant analysis, the investigated and the control groups were matched for age and BMI, and cleared of outliers (see Section 4.6.3 Data filtering). The characteristics of the resultant groups are presented in Table 10.

Fecal samples were used for the cultivation of various microorganisms. 1 g aliquots of each sample were homogenized and resuspended in 9 mL of sterile 0.9% saline solution. From the obtained solution, a series of subsequent dilutions of the suspension in saline was prepared until a dilution of 10^−9^ was reached. The suspension aliquots were cultivated on a set of selective media (Supplementary Table S1) and incubated at 37 °C for 24–48 h in anaerobic and aerobic conditions, respectively. Each type of microbial colony was characterized macroscopically and microscopically and identified through mass spectrometry with MALDI-TOF MS, using the MALDI Biotyper system with Microflex (Bruker Daltonics Inc., Billerica, MA, USA). The preparation of the samples was carried out according to the standard procedures for the isolation of proteins, which were mainly ribosomal. The mass spectra were analyzed within a range of 2000 to 20,000 *m*/*z*. The MALDI Biotyper version 3.0 library and the MALDI Biotyper version 3.0 software were used for identification considering the scores 1.5–1.7 for the genus level and scores > 2 for the species level.

### 4.3. Real-Time qPCR Quantification of Bacterial DNA

#### 4.3.1. Study Group

The overall qPCR study population included 83 patients diagnosed with NAFLD and 49 volunteers from the control group. Samples were chosen according to their DNA concentration and total DNA yield required for the correct setting of the study. Two different fecal DNA extraction methods were used in this study: QIAamp Fast DNA Stool Mini Kit (FS) and QIAamp PowerFecal DNA Kit (PF) (Qiagen, Venlo, The Netherlands). Four patient groups were categorized based on the year of collection and the method of extraction (refer to Table 11). Within each group, the control and test groups were carefully matched in terms of age and BMI, while also excluding any outliers (see Section 4.6.3 on data filtering). The details of these groups are outlined in Table 12.

#### 4.3.2. PCR Reaction

A DNA amount of the *Lactobacillaceae*, *Enterobacteriaceae* families; genera *Bacteroides* sp., *Bifidobacterium* sp., *Ruminococcus* sp., *Coprococcus* sp., *Oscillibacter* sp., *Veillonella* sp., *Odoribacter* sp., *Dorea* sp., *Blautia* sp., *Streptococcus* sp., *Desulfovibrio* sp., *Roseburia* sp., *Collinsella* sp.; species *Faecalibacterium prausnitzii*, *Fusobacterium nucleatum*, *Akkermansia muciniphila*, *Clostridium leptum*, *Clostridium symbiosum*, *Enterococcus faecalis*, was evaluated using corresponding primers and TaqMan probes (Supplementary Table S2). All primer sets were evaluated for their specificity and sensitivity in real-time PCR using DNA isolated from strains (Center for Strategic Planning, Moscow, Russia) and purified bacterial DNA from the Leibniz collection (DSMZ, Leibniz, Germany). Standard curves were obtained for each primer set using serial 1:10 dilutions (from 10^6^ to 1 copy of 16S rRNA gene in reaction) of measured purified bacterial DNA from the aforementioned collections. The data were cross-validated using ddPCR QX200 (Bio-Rad, Hercules, CA, USA). Corresponding slope and interception factors were calculated for each standard curve using standard sample concentrations expressed in genome copies/μL.

Prior to the qPCR experiment, the samples’ DNA concentration was determined fluorometrically on the Qubit^®^ 4.0 Fluorometer (Thermo Fisher Scientific, Waltham, MA, USA) using the Qubit^®^ dsDNA BR Assay Kit. Each sample was then diluted in sterile purified water to a concentration of 1.0 ng/µL. Those diluted aliquots were used in real-time qPCR assays and were stored at 4 °C between plate runs to preserve the samples from degradation. All samples were checked for sufficient total bacterial DNA amount by amplification of the 16S rRNA V4 region using universal primers.

All qPCR reactions were carried out using the 5× PCR Master Mix (Center for Strategic Planning) containing hot-start Taq-polymerase and 17.5 mM MgCl_2_, under the conditions recommended by the manufacturer, with 400 nM of each primer and TaqMan probe in a volume of 25 μL. 96-well optical-grade PCR plates sealed with optical sealing tape (Bio-Rad) were used. Sterile water served as the no template control. Each plate contained reactions of one qPCR assay to minimize threshold-dependent biases. All PCR runs contained 5-point series of the standard sample 10-fold dilutions, which were used to set a correct threshold in each plate (~10% of the control sample end fluorescence to ensure the same cycles for the standard samples in all plates).

The qPCR program: 95 °C—15 min, 45 cycles: (95 °C—15 s, 60 °C *—30 s) on CFX96 Touch™ (Bio-Rad).

### 4.4. Preparation of DNA Libraries and Sequencing

DNA extraction was conducted on samples from patients, as shown in Table 13.

DNA was extracted using QIAamp^®^ PowerFecal Kit (Qiagen) according to the manufacturer’s instructions. A dsDNA HS Assay Kit and Qubit^®^ 4.0 fluorometer (Thermo Fisher Scientific) were used to measure the DNA concentration, and the quality of isolated DNA was analyzed through electrophoresis in 1% agarose gel.

DNA libraries were prepared using PCR amplification with gene-specific primers for the V3–V4 regions of 16S rRNA. The following processes were performed in accordance with Illumina instructions. The quality of the prepared libraries was analyzed on the Bioanalyzer 2100 (Agilent Technologies, Santa Clara, CA, USA) using the High Sensitivity DNA kit (Agilent Technologies). A Qubit dsDNA HS Assay Kit and Qubit^®^ 4.0 fluorometer were used to measure the DNA concentration. A MiSeq Reagent Kit V2 Nano was used to prepare the DNA for sequencing; then, the samples were sequenced using the Illumina MiSeq platform (Illumina, San Diego, CA, USA) according to the manufacturer’s protocol, using reagents for double-ended reading and a read length of at least 250 bp. The amount of PhiX Control v3 was not less than 1%.

### 4.5. Metagenome Data Processing and Analysis

The Quantitative Insights in Microbial Ecology 2 (QIIME2), version 2022.2.0, was used to analyze and process the obtained sequencing reads [68]. Using Cutadapt plugin implemented in the QIIME2 toolkit, we excised the remnant primers (flanking the V3–V4 region), which could result as a contaminant after automatic Illumina post-processing [69]. Then, using DADA2 [70], reads were trimmed, filtered, overlapped, and combined into similarity cohorts to create a table of all amplicon sequence variants (ASVs) found within every sample. These were followed by actual and relative ASV abundances. Then, we generated a midpoint-rooted phylogenetic tree with the FastTree plugin [71], using the prior multiple alignment of ASVs produced via MAFFT [72]. After that, we performed the ASVs classification step via two Naïve Bayes classifiers from the scikit-learn library. These were trained on the reference databases of 16S rRNA: SILVA (v.138.99) and RDP (v.11.5). The obtained classification tables were merged with ASV abundance data, and the next analysis was conducted on the family taxonomy level. Rarefaction curves displayed asymptotic behavior per group, indicating that a 19,000 sampling depth was sufficient to avoid sampling errors (Supplementary Figure S1).

### 4.6. Statistical Analysis

#### 4.6.1. Software

All calculations were performed using R programming language (v 4.2.1) with data processing environment Rstudio 2022.02.3 (build 492).

#### 4.6.2. Data Conversion

(1) MALDI-TOF mass spectra profiles and the PCR data of every sample were transformed in accordance with the following rule:

- all zero elements were retained,
- non-zero elements were converted using a formula (Formula (1)):

(1) $$v_{ij}=\log_{10}x_{ij}$$

where $x_{ij}$—original non-zero value located in the *i*-th row, in the *j*-th column, $v_{ij}$—converted non-zero value.

(2) Custom normalization was performed for the 16S NGS data for the sparse taxa (after aggregation to the family level). This step was necessary to avoid sampling biases in the abundance of sparsely inhabited family groups. With the goal of minimizing sampling effects, low abundance taxonomic groups were ranked according to the following rule: all values in the ASV frequency table below 31 were replaced with 0; those from 31 to 99 were replaced with 75, and those from 100 to 149 were replaced with 125. Values outside these ranges remained unchanged. The NGS data were transferred to the phyloseq format (using the R package “phyloseq”). This package uses a specialized S4 class system to store all related the data as a single experiment-level object, which simplifies information exchange and analysis reproduction.

#### 4.6.3. Data Filtering

To exclude the influence of age, sex, and BMI on the differences in microbiome composition, the samples for each analysis were balanced according to these parameters. Two techniques were implemented to check the balance of covariates: principal component analysis (PCA) using the “factoextra” package, and nonparametric preprocessing for parametric causal inference using the “MatchIt” package [73] in R. First, samples with abnormal values were identified using PCA, and the position of healthy and diseased groups relative to each other (based on their BMI, age, and sex) was also assessed. For the calculations, we used the prcomp() R function of the “factoextra” package with the default parameters). Then, using the “CEM” method (coarsened exact matching) from the “MatchIT” package, the parameters were averaged between the groups by removing the outliers. Selected samples are presented in the subsequent analysis.

#### 4.6.4. Exploratory and Statistical Data Analysis

##### Mann–Whitney Test

A nonparametric Mann–Whitney test was performed with the “stats” library for bacterial content using all three methods (culture, NGS, and qPCR) and NGS alpha diversity data. CoDA transformation was chosen for the analysis of NGS data. To control the false discovery rate, all obtained p-values were corrected according to the Benjamini–Yekutieli procedure. The “stats” library was also used to implement this step. CoDA transformation was chosen for the analysis of NGS data and the alpha diversity for NGS data was also evaluated using the same statistical criterion.

##### Principal Coordinate Analysis

The principal components values and total scatter proportion of the original data were calculated using the prcomp() function of the “factoextra” library with default parameters for qPCR and MALDI-TOF data. The ordinate() function of the “phyloseq” library was used for NGS data. The first two components were visualized on the graph to compare the control and NAFLD groups.

##### Permutational Multivariate Analysis of Variance

PERMANOVA analysis of variance using the “vegan” package was performed. The result was achieved by separating the sum of squares for components within and between clusters using the centroid concept. We used this method to compare people from the control group and people with NAFLD, and to test the hypothesis that centroids and group variance are equivalent for all studied groups.

##### Latent Dirichlet Distribution Analysis

Latent Dirichlet distribution analysis was applied to detect differences between groups that were not in separately represented taxa, but in groups of bacteria that had tendencies to occur together (similar functions or inducing external factors). The FindTopicsNumber function in the “ldatuning” package in R was used to select the number of groups. The “MicrobiomStat” package was used to identify significant groups, specifying the p-value refinement using FDR methodology and a significance threshold of α = 0.05.

##### Penalized Regression

Microbial signatures in cross-sectional studies were identified using the “coda4microbiome” package. The model performs variable selection through penalty regression on the set of all pairwise logarithmic relationships of the binary trait (control and NAFLD). The analysis was performed with default parameters. The results were expressed as a (weighted) balance between the two taxon groups.

##### Simulated Data Synthesis

Synthetic data were used to increase the accuracy of the machine learning model. This technique is often used to keep the data confidential [74]; the same approach to increase the predictors for the correct construction of the model was utilized. The “R synthpop” package was used to implement this step.

##### Machine Learning Model

The model was created using the “H2O” package, and the results were interpreted using the DALEX package. The data were divided into test and training samples as a 70/30 ratio.

##### Correlation Scores

The correlation for the sample was estimated using the cor() function of the “stats” package; Spearman’s method was used to calculate it as data do not have a normal distribution. The results of the function were visualized using the “corrplot” package.

## 5. Conclusions

We used three independent methods (MALDI-TOF-MS, qPCR, and 16S NGS sequencing) to assess the differences in gut microbiota composition between NAFLD patients and a control group. The most interesting observations included enrichment in *Collinsella* sp. and *Oscillospiraceae* for the control samples and enrichment in *Lachnospiraceae* (and in particular *Dorea* sp.) and *Veillonellaceae* in NAFLD. The families, *Bifidobacteriaceae*, *Lactobacillaceae*, and *Enterococcaceae* (particularly *Enterococcus faecium* and *Enterococcus faecalis*), were also found to be important members of gut microbiota in NAFLD, but require further investigation due to the discrepancy of results in the analytical methods that were used.

## Figures and Tables

**Table 1:** Mann–Whitney results for MALDI-TOF-MS data.

List of Bacterial Species	Adjusted *p*-Value	Abundance
*Bacteroides uniformis*	0.021	Control
*Enterococcus faecium*	0.018	Control
*Collinsella aerofaciens*	0.011	Control
*Candida krusei*	0.045	NAFLD

**Table 2:** Bacteria showing similar correlations in the control and NAFLD samples for the PF group (data represent Spearman’s correlation coefficient).

	Control	NAFLD
*Bacteroides* sp.: *Oscillibacter* sp.	−0.45	−0.52
*Blautia* sp.: *Clostridium symbiosum*	0.67	0.48
*Blautia* sp.: *Dorea* sp.	0.39	0.72
*Blautia* sp.: *Streptococcus* sp.	0.41	0.53
*Clostridium symbiosum*: *Dorea* sp.	0.31	0.63
*Fusobcaterium nucleatum*: *Veillonella* sp.	0.51	0.4

**Table 3:** Bacteria showing similar correlations in the control and NAFLD samples for the FS group (data represent Spearman’s correlation coefficient).

	Control	NAFLD
Age: *Coprococcus* sp.	−0.3	−0.59
BMI: *Enterococcus faecalis*	0.61	0.41
BMI: *Lactobacillaceae*	0.43	0.5
*Akkermansia muciniphila*: *Fusobacterium nucleatum*	−0.5	−0.39
*Bacteroides* sp.: *Odoribacter* sp.	0.77	0.53
*Bacteroides* sp.: *Parabacteroides* sp.	0.6	0.62
*Bifidobacterium* sp.: *Blautia* sp.	0.38	0.56
*Bifidobacterium* sp.: *Clostridium symbiosum*	0.72	0.3
*Bifidobacterium* sp.: *Coprococcus* sp.	0.6	0.52
*Bifidobacterium* sp.: *Roseburia* sp.	0.67	0.26
*Bifidobacterium* sp.: *Ruminococcus* sp.	0.5	0.37
*Blautia* sp.: *Roseburia* sp.	0.63	0.43
*Blautia* sp. *Collinsella* sp.	0.52	0.61
*Blautia* sp.: *Clostridium symbiosum*	0.51	0.47
*Blautia* sp.: *Ruminococcus* sp.	0.47	0.5
*Blautia* sp.: *Dorea* sp.	0.8	0.64
*Clostridium symbiosum*: *Faecalibacterium prausnitzii*	0.68	0.34
*Clostridium symbiosum*: *Fusobacterium nucleatum*	−0.59	−0.25
*Clostridium symbiosum*: *Oscillibacter* sp.	0.51	0.37
*Clostridium symbiosum*: *Roseburia* sp.	0.64	0.63
*Clostridium symbiosum*: *Ruminococcus* sp.	0.63	0.29
*Collinsella* sp.: *Streptococcus* sp.	0.7	0.42
*Coprococcus* sp.: *Clostridium symbiosum*	0.58	0.49
*Coprococcus* sp.: *Roseburia* sp.	0.63	0.42
*Dorea* sp.: *Clostridium symbiosum*	0.58	0.64
*Dorea* sp.: *Collinsella* sp.	0.5	0.76
*Dorea* sp.: *Coprococcus* sp.	0.5	0.59
*Dorea* sp.: *Odoribacter* sp.	−0.29	−0.5
*Dorea* sp.: *Roseburia* sp.	0.81	0.52
*Desulfovibrio* sp.: *Enterococcus faecalis*	0.52	0.45
*Enterococcus faecalis*: *Streptococcus* sp.	0.33	0.69
*Faecalibacterium prausnitzii*: *Roseburia* sp.	0.39	0.65
*Lactobacillaceae*: *Ruminococcus* sp.	0.3	0.77
*Lactobacillaceae*: *Streptococcus* sp.	0.71	0.54
*Odoribacter* sp.: *Parabacteroides* sp.	0.75	0.8
*Roseburia* sp.: *Streptococcus* sp.	0.39	0.58
*Ruminococcus* sp.: *Roseburia* sp.	0.52	0.5
*Ruminococcus* sp.: *Streptococcus* sp.	0.34	0.71

**Table 4:** Mann–Whitney results for RDP data (Family).

Domain	Phylum	Class	Order	Family	Adjusted *p*-Value	Abundance
Bacteria	Firmicutes	Clostridia	Clostridiales	*Gracilibacteraceae*	0.017	NAFLD
Bacteria	Firmicutes	Negativicutes	Acidaminococcales	*Acidaminococcaceae*	0.034	control
Bacteria	Bacteroidetes	Chitinophagia	Chitinophagales	*Chitinophagaceae*	0.038	NAFLD
Bacteria	Planctomycetes	Planctomycetacia	Pirellulales	*Pirellulaceae*	0.044	NAFLD
Bacteria	Firmicutes	Erysipelotrichia	Erysipelotrichales	*Erysipelatoclostridiaceae*	0.046	NAFLD
Bacteria	Bacteroidetes	Bacteroidia	Bacteroidales	*Muribaculaceae*	0.048	NAFLD
Bacteria	Proteobacteria	Betaproteobacteria	Burkholderiales	*Comamonadaceae*	0.048	NAFLD

**Table 5:** Mann–Whitney results for SILVA data (Family).

Domain	Phylum	Class	Order	Family	Adjusted *p*-Value	Abundance
Bacteria	Firmicutes	Negativicutes	Acidaminococcales	*Acidaminococcaceae*	0.035	control

**Table 6:** Statistically significant taxonomic groups for several methods of analysis used.

	NGS 16S	qPCR	Culture
*Collinsella* sp.	Enrichment in control (discovered in 38–48% of samples, content less than 0.5%)	Statistically significant taxon according to the Mann–Whitney method (PF group)	Enrichment in control (discovered in 38–48% of samples, content less than 0.5%)
*Fusobacterium nucleatum*	Not presented—discovered in 2 patients (content less than 0.05%)	Statistically significant taxon according to the Mann–Whitney method (FS group) An important taxon in XGBoost analysis: PF group—#7 FS group—#2 Enrichment in NAFLD	Not presented
*Acidaminococcaceae*	An important taxon in the latent Dirichlet distribution analysis (SILVA) An important taxon in Linear Discriminant Analysis (SILVA, RDP) Statistically significant taxon according to the Mann–Whitney method (SILVA, RDP) Enrichment in control	Not analyzed	Not presented—discovered only in 1 patient
*Bifidobacteriaceae*	An important taxon in the latent Dirichlet distribution analysis (SILVA) Enrichment in control	An important taxon in XGBoost analysis *Bifidobacterium* sp. (#4, PF group) Enrichment in NAFLD	Important taxa in XGBoost analysis: *Bifidobacterium adolescentis* (#1) *Bifidobacterium longum* (#2) An important taxon in Principal Component Analysis: *Bifidobacterium adolescentis* Enrichment in control
*Enterococcaceae*	An important taxon in Penalty Regression analysis (SILVA) An important taxon in Linear Discriminant Analysis (SILVA, RDP) Enrichment in control (Discovered in 17–21% of samples, content less than 0.5%) *E. faecalis*—discovered only in 3 patients (content less than 0.1%)	Statistically significant taxon according to the Mann–Whitney method (PF2020 group): *E. faecalis* Enrichment in NAFLD	Statistically significant taxon according to the Mann–Whitney method *Enterococcus faecium* An important taxon in XGBoost analysis: *Enterococcus faecium* (#5) Enrichment in control An important taxon in XGBoost analysis: *Enterococcus faecalis* (#4) Enrichment in control
*Lachnospiraceae*	An important taxon in the latent Dirichlet distribution analysis (SILVA, RDP) Enrichment in NAFLD	Important taxa in XGBoost analysis (PF group): *Dorea* sp. (#8) *Roseburia* sp. (#9) Statistically significant taxon according to the Mann–Whitney method *Dorea* sp. (FS group) All family representatives analyzed (*Blautia* sp., *Dorea* sp., *Roseburia* sp.) are enriched in NAFLD	Not presented—discovered only in 1 patient
*Lactobacillaceae*	An important taxon in penalty regression analysis (RDP) Enrichment in control	Statistically significant taxon according to the Mann–Whitney method (PF20 group) Enrichment in NAFLD	An important taxon in XGBoost analysis: *Lactobacillus plantarum* (#3) *Lactobacillus delbrueckii* (#6) An important taxon in Principal Component Analysis: *Leuconostoc lactis* Enrichment in control
*Muribaculaceae*	An important taxon in the latent Dirichlet distribution analysis (RDP) Statistically significant taxon according to the Mann–Whitney method (RDP) Enrichment in NAFLD	Not analyzed	Not presented
*Oscillospiraceae*	An important taxon in the latent Dirichlet distribution analysis (SILVA) Enrichment in control	Important taxa in XGBoost analysis (PF group): *Ruminococcus* sp. (#1) *Faecalibacterium prausnitzii* (#7) All family representatives analyzed (*Faecalibacterium prausnitzii*, *Oscillibacter* sp., *Ruminococcus* sp.) are enriched in control	Not presented
*Ruminococcaceae*	An important taxon in the latent Dirichlet distribution analysis (SILVA, RDP) An important taxon in penalty regression analysis (RDP) Enrichment in control (RDP), in NAFLD (SILVA)	An important taxon in XGBoost analysis: *Ruminococcus* sp. (#1, group PF) Enrichment in control	Not presented—discovered only in 1 patient
*Veillonellaceae*	An important taxon in the latent Dirichlet distribution analysis (RDP) Family—Enrichment in NAFLD *Veillonella* sp.—slight enrichment in control (content less than 0.5%)	Statistically significant taxon according to the Mann–Whitney method (group PF21) *Veillonella* sp. Enrichment in control	Not presented—discovered only in 3 patients

**Table 7:** Matching correlations for the PF and FS groups. (Data represent Spearman’s correlation coefficient).

	PF Control	FS Control	PF NAFLD	FS NAFLD	References
*Bacteroides* sp.: *Parabacteroides* sp.	0.35	0.6	0.46	0.62	[46,47,48]
*Blautia* sp.: *Clostridium symbiosum*	0.67	0.51	0.48	0.47	-
*Blautia* sp.: *Dorea* sp.	0.39	0.8	0.72	0.64	[29,46,49,50]
*Dorea* sp.: *Clostridium symbiosum*	0.31	0.58	0.63	0.64	-
*Dorea* sp.: *Collinsella* sp.	0.43	0.5	0.32	0.76	[51,52,53]
*Lactobacillaceae*: *Streptococcus* sp.	0.43	0.71	0.4	0.54	[54,55,56]
*Blautia* sp: *Collinsella* sp.	0.34	0.52			-
*Collinsella* sp.: *Streptococcus* sp.	0.34	0.7			[57,58]
*Fusobacterium nucleatum*: *Veillonella* sp.	0.51	0.49			[59,60]
*Odoribacter* sp.: *Parabacteroides* sp.	0.39	0.75			[61,62]
*Bifidobacterium* sp.: *Collinsella* sp.			0.35	0.78	[24,58,63,64]
*Blautia* sp.: *Streptococcus* sp.			0.53	0.52	[54,65,66]

**Table 8:** Matching correlations for qPCR and culture method.

	Control	NAFLD
qPCR *E. faecalis*—Culture *E. faecalis*	0.41	0.33
qPCR *Enterobacteriaceae*—Culture *Enterobacteriaceae*	0.38	0.48
qPCR *Enterobacteriaceae*—qPCR *E. faecalis*	0.43	0.08
Culture *Enterobacteriaceae*—Culture *E. faecalis*	0.35	0.32

**Table 9:** The main characteristics of the study subjects.

Parameter	NAFLD Patients (n = 155)	Controls (n = 44)
Sex: female, n (%)	91 (58.7)	34 (77.3)
Age, years, median (IR)	52 (44–60)	47 (36–51.8)
BMI, kg/m^2^, median (IR)	30.5 (28.1–33.8)	23.4 (21.5–26.4)
Normal weight (BMI < 25 kg/m^2^), n (%)	11 (7.1)	26 (59.1)
Overweight (25 ≤ BMI < 30 kg/m^2^), n (%)	56 (36.1)	17 (38.6)
Obesity (BMI ≥ 30 kg/m^2^), n (%)	88 (56.8)	1 (2.3)
Waist circumference, cm, median (IR)	101 (93–109)	79.5 (70.4–89.8)
Hip circumference, cm, median (IR)	111.5 (105–120)	99 (94.8–103.5)
Epicardial fat, mm, median (IR)	11 (8–35)	7.5 (4.3–9.8)
Platelet count, 10^9^/L, median (IR)	236 (202–278)	233 (205–255)
FLI, median (IR)	74 (60.8–91.3)	11 (5.5–32.5)
ALT, IU/L, median (IR)	24 (17–37)	14 (10–24)
AST, IU/L, median (IR)	21 (17–26)	18 (15.5–23)
GGT, IU/L, median (IR)	30 (20.3–49.8)	17 (13–30)
Total bilirubin, mg/dL, median (IR)	13 (10–18)	11.6 (8.8–14.1)
Glucose, mmol/L, median (IR)	5.7 (5.3–6.3)	5.3 (5.03–5.8)
Total cholesterol, mg/dL, median (IR)	5.4 (4.6–6.3)	5.3 (4.5–6.5)
Triglycerides, mg/dL, median (IR)	1.44 (1.03–2.04)	0.92 (0.68–1.3)
Fibrinogen, g/L, median (IR)	3.8 (3.4–4.2)	3.5 (3.2–4.0)
CRP, mg/L, median (IR)	1.8 (0.9–3.3)	0.88 (0.43–1.7)
Creatinine, μmol/L, median (IR)	75 (69–87)	70 (63.3–79.8)
Uric acid, μmol/L, median (IR)	5.8 (4.9–7.0)	4.6 (4.0–5.9)
Insulin, μIU/mL, median (IR)	11.7 (8.2–15.2)	6.3 (4.9–8.1)
HOMA-IR > 2.7, n (%)	86 (55.4)	7 (15.9)
TyG, median (IR)	4.7 (4.6–4.9)	4.6 (4.4–4.8)
Fibrotest, median (IR)	0.16 (0.10–0.22)	0.12 (0.08–0.21)

The presented values denote frequency (%) or median (interquartile range). BMI: body mass index; FLI: fatty liver index; ALT: alanine aminotransferase; AST: aspartate aminotransferase; GGT: gamma-glutamyl transferase; CRP: C-reactive protein; HOMA-IR: homeostasis model assessment of insulin resistance; TyG: triglyceride glucose index.

**Table 10:** Baseline characteristics of patients for MALDI-TOF-MS analysis.

	Control Group (n = 38)	NAFLD Group (n = 38)
Age, years Average ± SD [min; max]	46 ± 8.6 [34; 65]	50.3 ± 8.5 [34; 67]
BMI, kg/m^2^ Average ± SD [min; max]	26.8 ± 3.1 [23.3; 33.9]	27.3 ± 2.4 [23.6; 33.6]
Sex, male, n (%)	46	50

**Table 11:** Baseline characteristics of groups for qPCR analysis.

	Group PF20	Group FS20	Group PF21	Group PF21+
Collection year	2020	2020	2021	2021
Extraction method	PF	FS	PF	PF
Samples	NAFLD and Control	NAFLD and Control	NAFLD and Control	NAFLD
Peculiarities	-	-	-	Higher BMI, mostly women (see Table 12)

**Table 12:** Baseline characteristics of samples for qPCR analysis.

	Control Group			NAFLD Group			
Subgroup #	1 (n = 23)	2 (n = 14)	3 (n = 15)	1 (n = 19)	2 (n = 14)	3 (n = 19)	4
Age, years Average ± SD [min; max]	46.2 ± 8.1 [32; 64]	47.2 ± 11 [31; 64]	45.3 ± 9.7 [30; 64]	52.9 ± 7.6 [35; 65]	51.8 ± 9.1 [36; 65]	47.7 ± 8.7 [34; 62]	50.3 ± 10.1 [27; 70]
BMI, kg/m^2^ Average ± SD [min; max]	27.7 ± 3.2 [22.7; 33.2]	24.6 ± 3.9 [19; 34]	25.3 ± 2.1 [21.5; 28.8]	28 ± 2.7 [22.5; 33.2]	27.3 ± 3.4 [21; 32]	26.7 ± 2 [23.5; 30.1]	34.2 ± 4.1 [29.5; 46.1]
Sex, male, n (%)	87	71.4	53.3	42.1	71.4	57.9	13.5

**Table 13:** Baseline characteristics of groups for NGS analysis.

	Control Group (n = 29)	NAFLD Group (n = 29)
Age, years Average ± SD [min; max]	45.6 ± 8.1 [31; 64]	51 ± 8.2 [34; 65]
BMI, kg/m^2^ Average ± SD [min; max]	27.3 ± 2.9 [22.9; 33.2]	27.5 ± 2.5 [22.5; 33.2]
Sex, male, n (%)	79	48

## Data Availability

The datasets used and analyzed in the present study are available from the corresponding author on reasonable request.

## Supplementary Materials

The following supporting information can be downloaded at: https://www.mdpi.com/article/10.3390/ijms242015272/s1.
