# Supplementary material for: Gut Microbiota Patterns in Patients with Non-Alcoholic Fatty Liver Disease: A Comprehensive Assessment Using Three Analysis Methods
Source: Int J Mol Sci. 2023 Oct 17;24(20):15272. doi: 10.3390/ijms242015272 (PMC10607775; doi:10.3390/ijms242015272)

Supplementary 1. Table S1. Set of selective media were used to isolate microorganism.

Columbia Agar Base (Oxoid, UK)
Blood agar (Pronadisa Conda, Spain)
Endo Agar (Pronadisa Conda, Spain)
Salmonella Shigella Agar (Oxoid, UK)
Levine Agar (Pronadisa Conda, Spain)
Salmonella Chromogenic Agar (Pronadisa Conda, Spain)
Sabouraud Dextrose Agar w/Chloramphenicol (Oxoid, UK)
Bismuth Sulfite Agar (Wilson Blair)
Urinary Tract Infections Chromogenic Agar (Pronadisa Conda, Spain)
Mannitol Salt Agar (Himedia, India)
CLED Agar (Pronadisa Conda, Spain)
Enterococcus Selective Agar (Pronadisa Conda, Spain),
MRS Agar (De Man, Rogosa, Sharpe) (Pronadisa Conda, Spain)
Chocolate agar with growth factors (Obolensk, Russia)
Schaedler Agar (Oxoid, UK)
Anaerobic Agar (Oxoid, UK)
Reinforced Clostridial Agar (Oxoid, UK)
Wilkins Chalgren Medium (Pronadisa Conda, Spain)
Bifidobacterium Agar (Himedia, India)
Thioglycollate USP medium (Oxoid, UK)
Brain Heart Infusion Agar (Pronadisa Conda, Spain)

Supplementary 2. Table S2. Primer sets used in the qPCR study

Target organism	Primer set	Sequence (5′ to 3′)
Total bacteria amount	515F	GTGCCAGCAGCCGCGGTAA
	806R	GACTACCAGGGTATCTAATCC
	Uni probe Fam/BHQ1	TMTCTRMGCATTYCACCGCTAC
Akkermansia muciniphila	AKK mun F	ACAAGCGGTGGAGTATGTGG
	AKK mun R	CATGCAGCACCTGTGTAACG
	AKK mun P Fam/BHQ1	CGAAGAGTCGCATGCTTTCACATGTT
Bacteroides sp.	Bac F	ATTGGGTTTAAAGGGAGCGT
	Bac R	CTACACCACGAATTCCGCCT
	Bac P Fam/BHQ1	TCAGTTGTGAAAGTTTGCGGCTCAAC
Bifidobacterium sp.	Bif F	CGAACGGGTGAGTAATGCGT
	Bif R	TGATAGGACGCGACCCCATC
	Bif P Fam/BHQ1	CCGGAATAGCTCCTGGAAACGGGT
Blautia sp.	Blau F	AAGGAAGAAGTATCTCGGTATGTA
	Blau R	CAACGTCAGTTACCGTCCAG
	Blau P Fam/BHQ1	CTGGGTGTAAAGGGAGCGTAGACGG
Christensenellaceae	Christ F	ACCGCATGAGACCACGAAACCG
	Christ R	TCCCATAGGACAAAGGTTTAC
	Christ P Fam/BHQ1	TCGATGCAGGATGGGCTCGCG
Clostridium leptum	C.lept F	AAAGGAGCAATCCGCTGG
	C.lept R	CGGCTACCGATCGTCGCT
	C.lept P Fam/BHQ1	TGGACTCGCGTCCGATTAGCCA
Clostridium symbiosum	C.symb F	CTTATTCTAAGTAGCCAGCGG
	C.symb R	TAGCACGTGTGTAGCCCAGA
	C.symb P Fam/BHQ1	AACTCTTGGGAGACTGCCAGGG
Collinsella sp.	Coll F	CAGCCAACGCATTAAGCGT
	Coll R	TGTGCAGCCCAGGGCATAAG
	Coll P Fam/BHQ1	TGACTTGACGTCGTCCCCGCCCT
Coprococcus sp.	Copro F	GTTYCAGTAGCCAGCAKTAAGA
	Copro R	ACAGGTTCGCTTYTCTTTGTAACT
	Copro P Fam/BHQ1	GGCAGTCTSTCCAGAGTGCCCA
Desulfovibrio sp.	Des-vib F	CAACGATGGGTAGCCGAT
	Des-vib R	GCCGGTGCTTCCTTTGA
	Des-vib P Fam/BHQ1	GATCGGCCACACTGGAACTGAAAC
Dorea sp.	Dorea F	GCAGCTAACGCAATAAGCAG
	Dorea R	GAAAAGCTTCCATTACGAAGC
	Dorea P Fam/BHQ1	CTGATCTTGACATCCCGATGAC

Enterobacteriaceae	Ent-ceae F	TCCCCGGGCTCAACCTG
	Ent-ceae R	CCAARTCGACATCGTTTACG
	Ent-ceae P Fam/BHQ1	CTCTACRAGACTCWAGCYTGCCA
Enterococcus faecalis	Ent-fae F	GCAAGTCGAACGCTTCTTTC
	Ent-fae R	GCCTTTCACTCTTATGCCATG
	Ent-fae P Fam/BHQ1	CATAAACTGTTATGCGGTATTAGCACC
Faecalibacterium prausnitzii	F.prau F	AAGATAATGACGGTACYCAACAAG
	F.prau R	CACTTCCAACTTGTCTTCCCGC
	F.prau P Fam/BHQ1	CAACGCTTGTGACCTACGTTTTAC
Fusobacterium nucleatum	F.nuc F	ACTTAGCAAATCAAGAAGAAGCAAG
	F.nuc R	CATTTCAGCTTCTAATTTCTTTAAAGC
	F.nuc P Fam/BHQ1	ACAAGCACTAGCACAAAATGAACAAGTTT
Lactobacillaceae	Lac-ceae F	CAGCAGTAGGGAATCTTCCA
	Lac-ceae R	AGTTTCCGATGCRVTTC
	Lac-ceae P Fam/BHQ1	TGATGGAGCAACRCCGCGTG
Odoribacter sp.	Odor F	GCCCATGGAAACGTGGAT
	Odor R	CAGTCTTCCCTCACGCGA
	Odor P Fam/BHQ1	AGGGGTTCTGAGAGGAAGGTC
Oscillibacter sp.	Oscill F	TTAACTGCGGCACGCAG
	Oscill R	GTGTTAACTGCGGCACG
	Oscill P Fam/BHQ1	AGGTGTGCGGGGACTGACC
Parabacteroides sp.	Parab F	GACACTGAAGCACGAAAGC
	Parab R	CGGTCCAAATGCGTTCAAA
	Parab P Fam/BHQ1	TGGTAGTCCACGCAGTAAACGATGAT
Roseburia sp.	Rsb F	ATTGGAAACTGTCGTAC
	Rsb R	CGAAGAGCAATGCTCCCCG
	Rsb P Fam/BHQ1	CTGACGCTGAGKCTCGAAAG
Ruminococcus sp.	Rum F	TAGWKCTTGAGTGAAGTAGAGG
	Rum spp R	TCGWGCCTCAGCGTCAGTWAA
	Rum P Fam/BHQ1	ATMTCTACGCATTTCACCGCTACAC
Streptococcus sp.	Strep F	AGCGCAGGCGGTTAGATAAG
	Strep R	CCCCGGAAAGGGTCTAACAC
	Strep P Fam/BHQ1	AWAGTACGCTTTGGAAACTGTTTAACTTGAG
Veillonella sp.	Veill R	GTYTGGGCCGTGTCTCAGTC
	Veill F	GTAATCAACCTGCCCTTCAGAG
	Veill P Fam/BHQ1	TAAGCTATCRCTGAAGGAGGGGATTGC

Supplementary 3. Figure S1. Shannon diversity index rarefaction curves at various sequencing depths. All curves hit a plateau at the 5000 sequencing depth and demonstrated a similar pattern of diversity enrichment. Every group's evaluation of Shannon significance level started at the sequencing depth with a 0-diversity index.

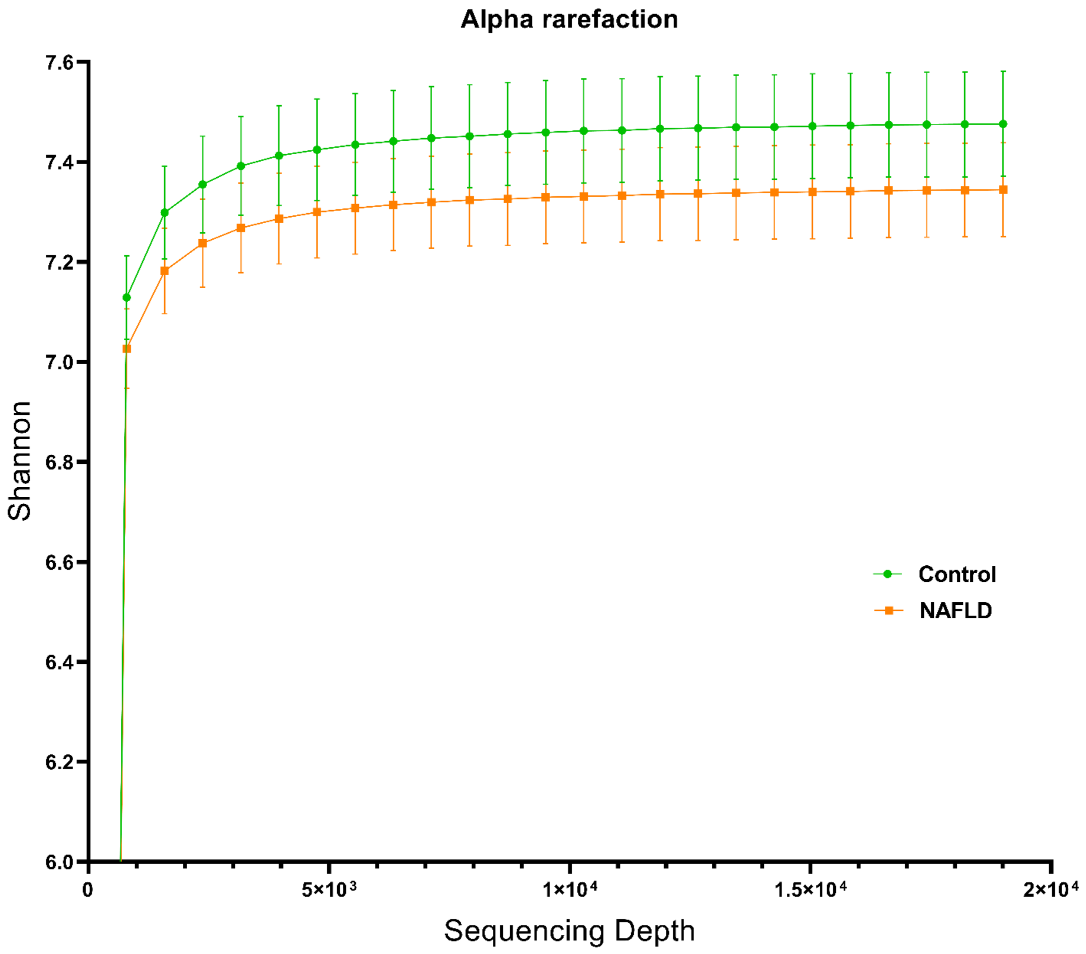

Supplementary 4. Figure S2. The significant predictors of XGBOOST model.

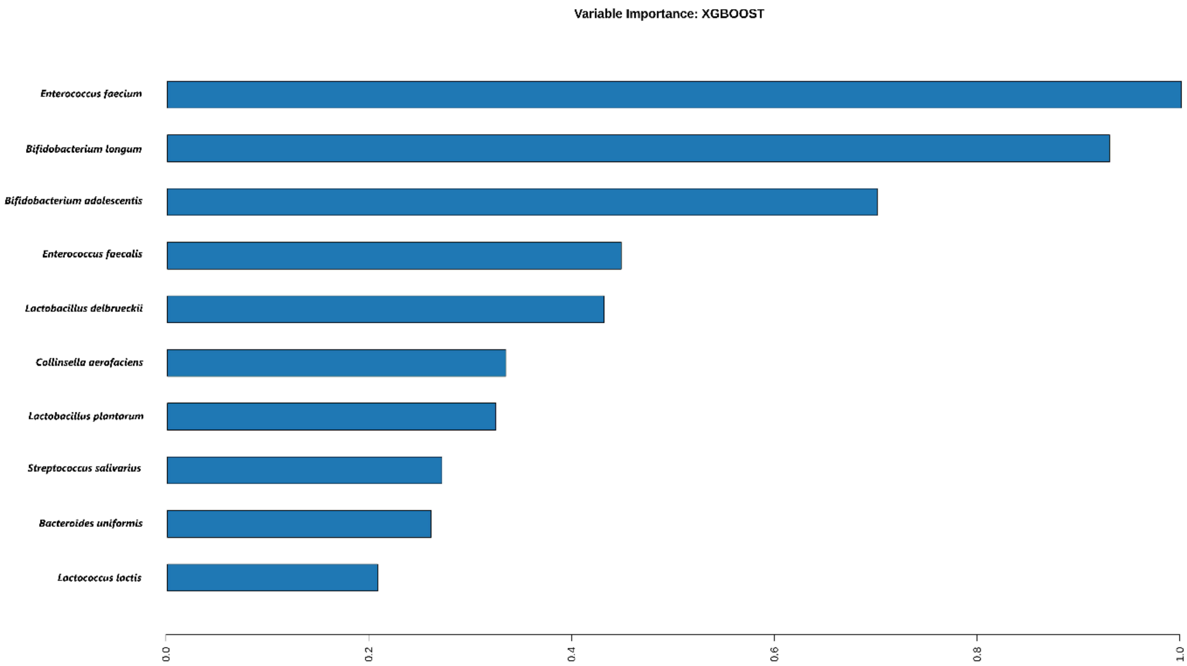

Supplementary 5. Figure S3. Top ten predictors for XGBOOST model (PF group).

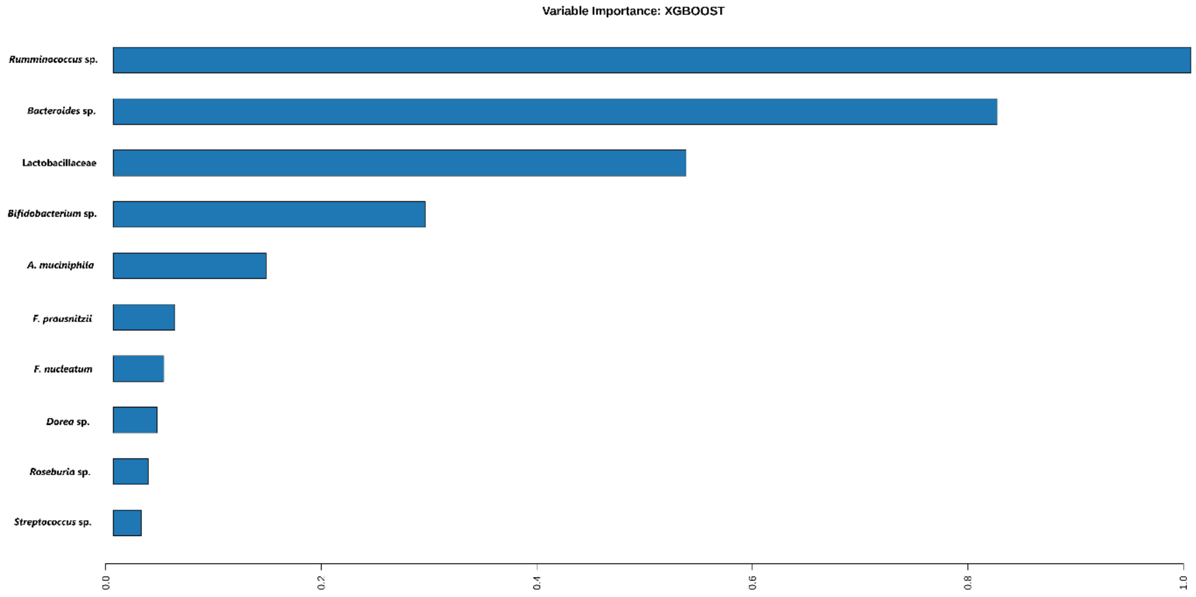

Supplementary 6. Figure S4. Top ten predictors for XGBOOST model (FS group).

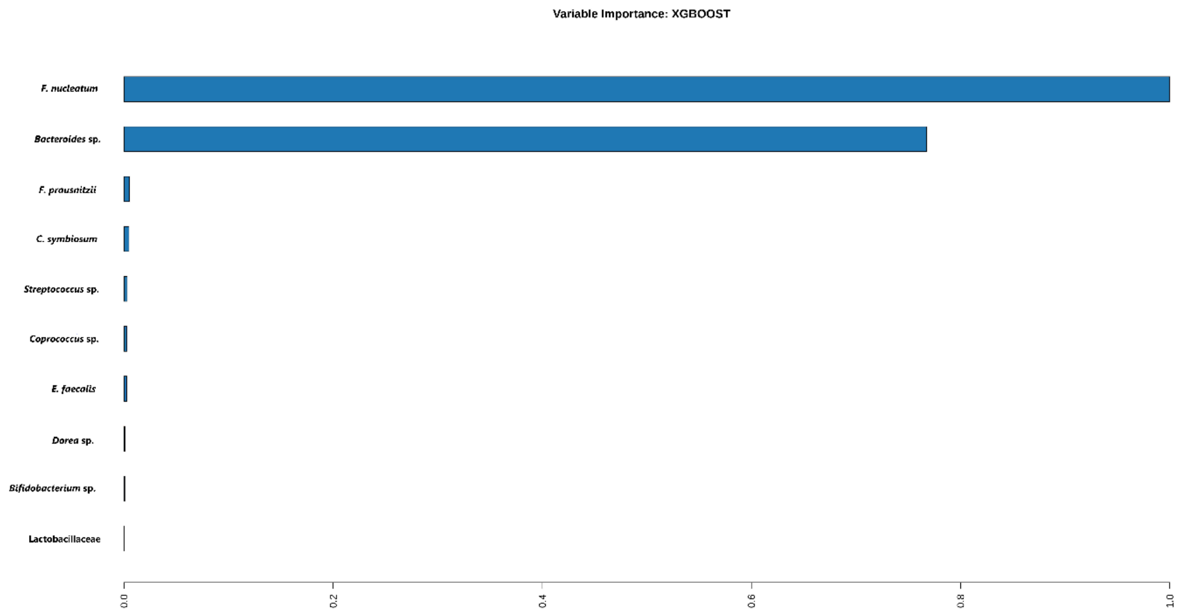

Supplementary 7. Figure S5. Correlation matrix for NAFLD samples (Groups PF and PF21+). Positive correlations are highlighted in red, negative correlations in blue (see the scale).

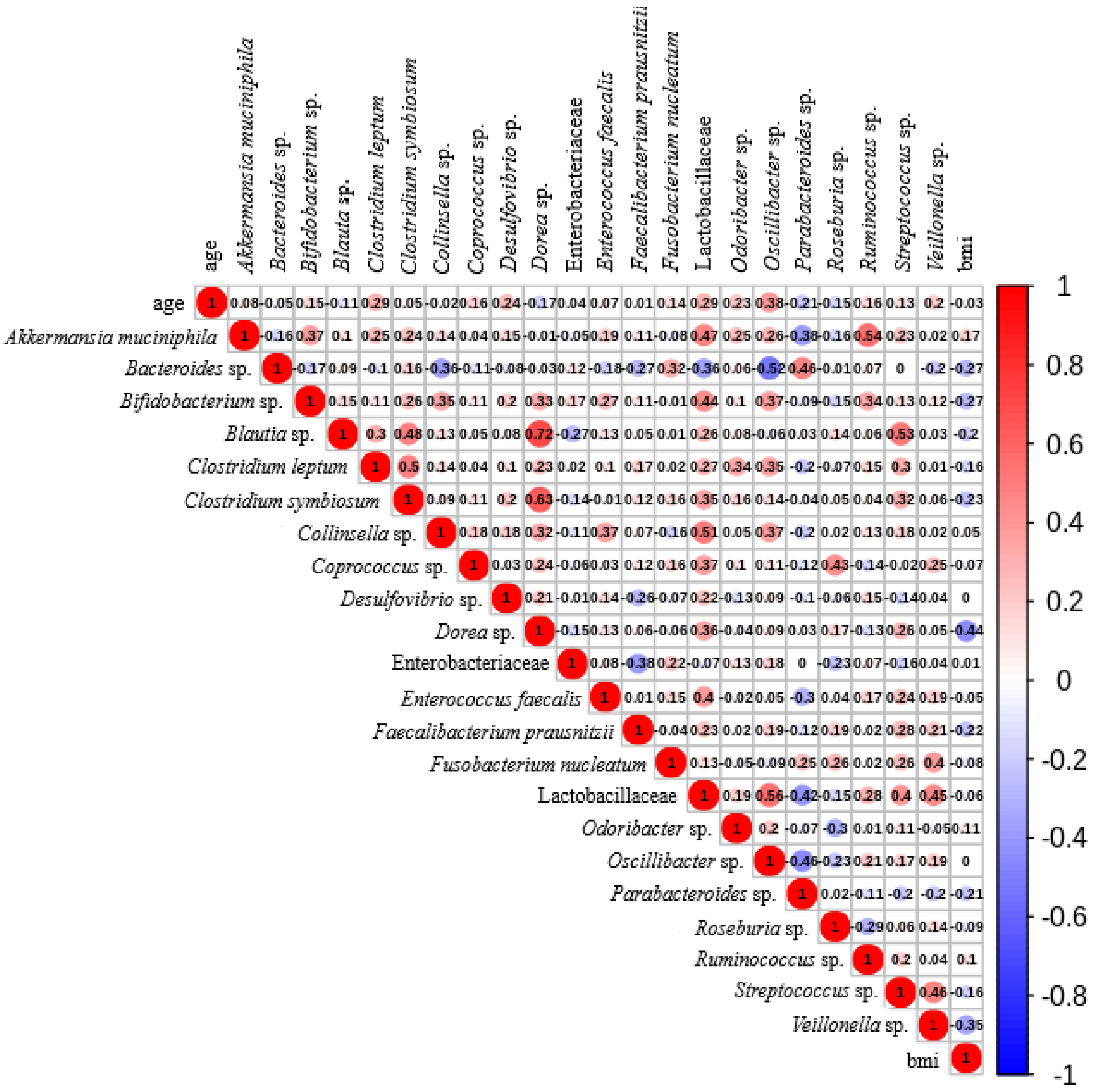

Supplementary 8. Figure S6. Correlation matrix for the Control sample (PF group).

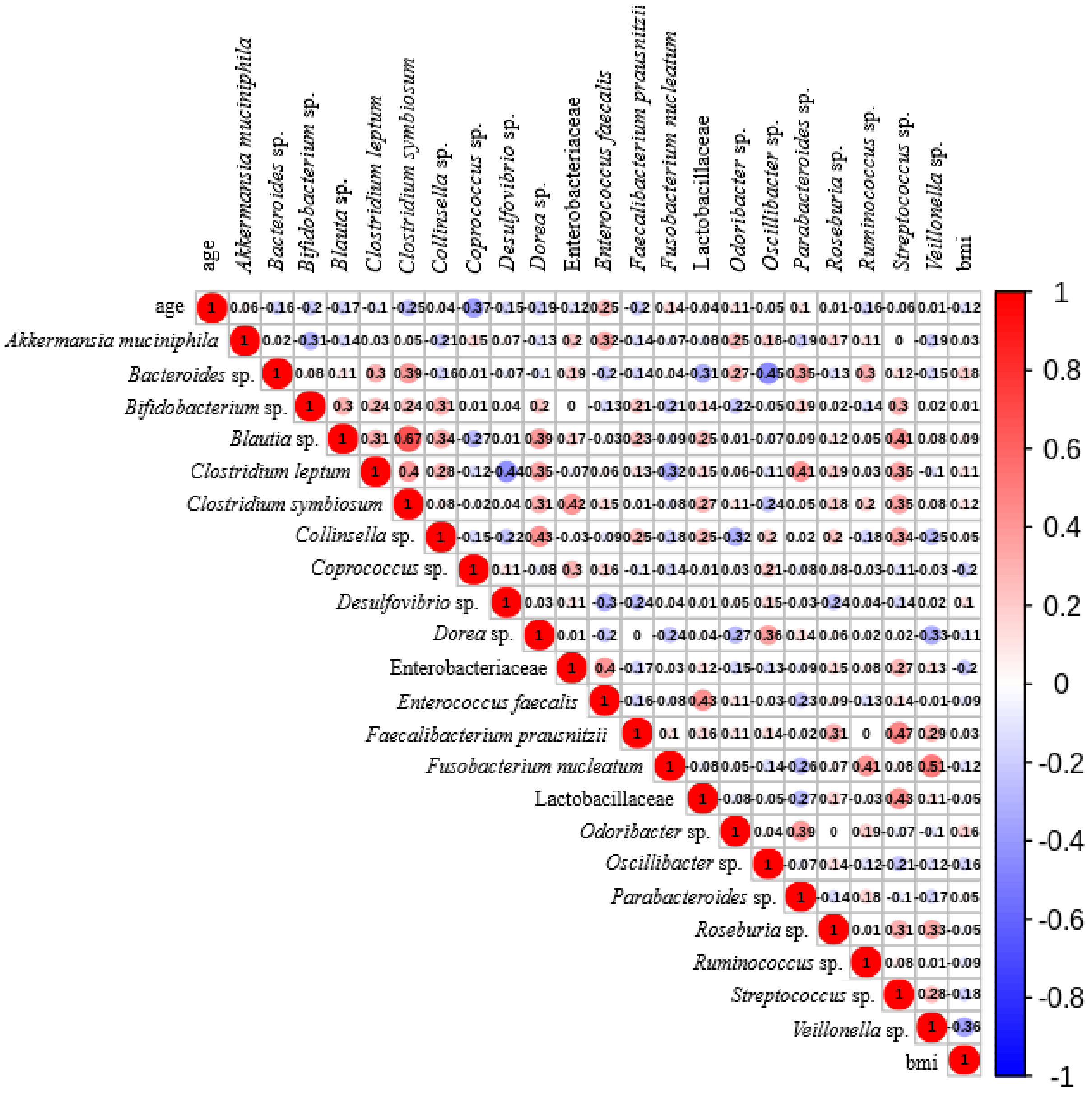

Supplementary 9. Figure S7. Correlation matrix for the NAFLD sample (FS group).

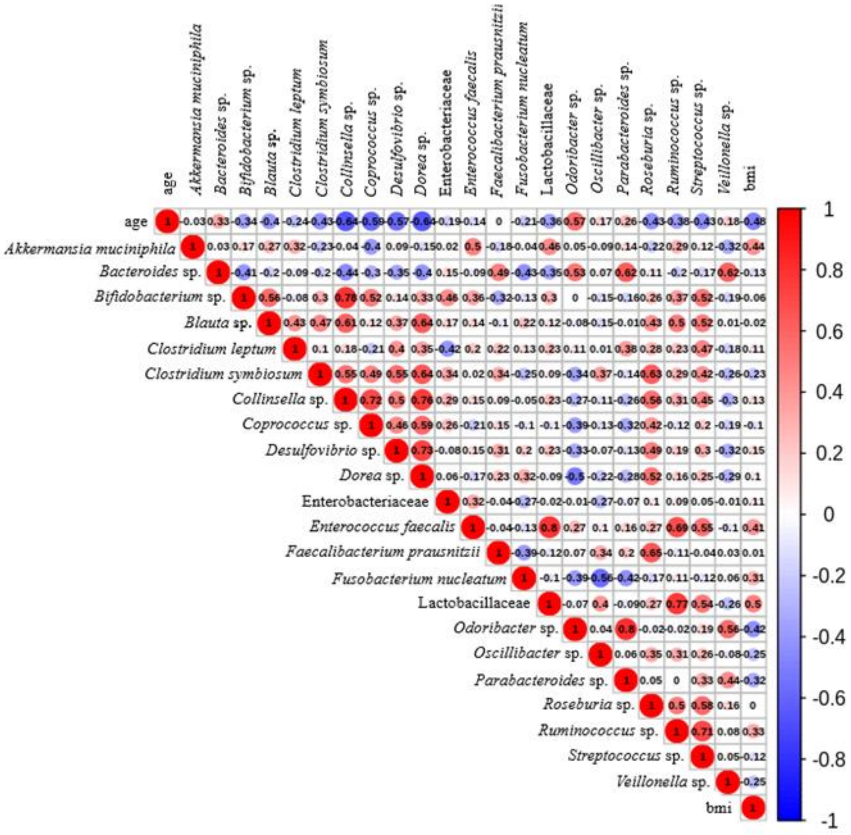

Supplementary 10. Figure S8. Correlation matrix for the Control sample (FS group).

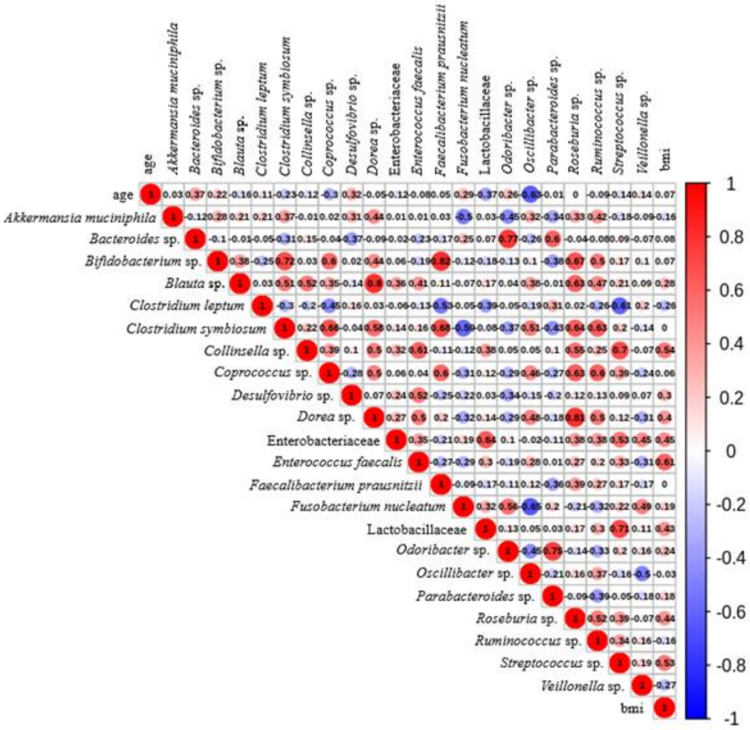

Supplement: Supplementary file 1 [file ijms-24-15272-s001.zip › ijms-2590119-supplementary.pdf]
